# Effect of the Crystal Environment on Side-Chain Conformational Dynamics in Cyanovirin-N Investigated through Crystal and Solution Molecular Dynamics Simulations

**DOI:** 10.1371/journal.pone.0170337

**Published:** 2017-01-20

**Authors:** Logan S. Ahlstrom, Ivan I. Vorontsov, Jun Shi, Osamu Miyashita

**Affiliations:** 1 Department of Molecular, Cellular, and Developmental Biology, University of Michigan, Ann Arbor, Michigan, United States of America; 2 Department of Chemistry and Biochemistry, University of Arizona, Tucson, Arizona, United States of America; 3 RIKEN Advanced Institute for Computational Science, Chuo-ku, Kobe, Hyogo, Japan; Hong Kong University of Science and Technology, HONG KONG

## Abstract

Side chains in protein crystal structures are essential for understanding biochemical processes such as catalysis and molecular recognition. However, crystal packing could influence side-chain conformation and dynamics, thus complicating functional interpretations of available experimental structures. Here we investigate the effect of crystal packing on side-chain conformational dynamics with crystal and solution molecular dynamics simulations using Cyanovirin-N as a model system. Side-chain ensembles for solvent-exposed residues obtained from simulation largely reflect the conformations observed in the X-ray structure. This agreement is most striking for crystal-contacting residues during crystal simulation. Given the high level of correspondence between our simulations and the X-ray data, we compare side-chain ensembles in solution and crystal simulations. We observe large decreases in conformational entropy in the crystal for several long, polar and contacting residues on the protein surface. Such cases agree well with the average loss in conformational entropy per residue upon protein folding and are accompanied by a change in side-chain conformation. This finding supports the application of surface engineering to facilitate crystallization. Our simulation-based approach demonstrated here with Cyanovirin-N establishes a framework for quantitatively comparing side-chain ensembles in solution and in the crystal across a larger set of proteins to elucidate the effect of the crystal environment on protein conformations.

## Introduction

Protein side-chain conformations observed by X-ray crystallography play a key role in understanding biological function, such as catalysis and molecular recognition, and in identifying lead compounds during drug design. However, side-chain conformational dynamics that are important for these processes may remain unclear, as X-ray structures depict the large majority of side chains in a single conformation, which is under the influence of the crystal environment. Thus, side-chain conformations from X-ray data must be carefully interpreted.

Side-chain conformations may vary across different crystal structures of the same protein. Comparison of chemically identical proteins in different crystal forms showed notable differences in side-chain conformation for residues near crystal packing interfaces compared to residues farther way from these regions, especially for long, polar and charged side chains [1]. Subsequent analysis of a larger dataset of proteins revealed roughly the same level of side-chain structural variability for both contacting and non-contacting residues [2]. Consistent with this observation, including crystal neighbors in a side-chain prediction algorithm only moderately improved the accuracy of prediction [3], while incorporating longer-range electrostatic and solvation effects improved performance [1, 3]. Side-chain conformations observed in X-ray models are also sensitive to refinement methods and crystallization conditions [1, 4].

Moreover, the restriction of conformational dynamics of side chains on the protein surface upon crystallization may disfavor the formation of packing interfaces. This effect is the basis of the surface-entropy reduction (SER) method [5, 6] in which longer side chains on the protein surface are mutated to shorter ones to minimize entropy loss during protein crystallization. The degree of side-chain dynamics in the crystal is commonly inferred from thermal factors. Comparison of thermal factors in 25 non-isomorphous crystal structures of T4 lysozyme suggested that side-chain mobility in the crystal is representative of the solution state [7]. However, due to diffraction resolution limits it is typical that side chains on the protein surface are modeled in a single or, at most, two alternative conformations during crystal structure refinement. In addition, there is evidence that cryocooling for X-ray data collection can remodel side-chain conformational ensembles for both solvent-exposed and buried residues compared to room-temperature crystals [8]. Thus, it is valuable to quantitatively assess side-chain conformational dynamics in solution and in the crystal. Molecular dynamics (MD) simulation presents a powerful complimentary approach to address this issue.

Comparison of crystal and solution MD simulations has yielded key insights into the effect of the crystal environment on side-chain conformational dynamics. For example, including the crystalline environment in addition to solvent effects during simulation of bovine pancreatic trypsin inhibitor (BPTI) improved agreement between side-chain torsion potentials and observed X-ray conformations [9]. Additional simulations of BPTI in solution and in the crystal revealed notable variation in side-chain conformation for polar residues [10]. Furthermore, simulations on the streptavidin-biotin complex showed good agreement for side-chain χ_1_ angles in solution and in the crystal, and indicated that the solvent composition of the crystal environment may influence side-chain conformation [11]. More recent work focusing on MD force-field validation suggested a similar degree of conformational disorder for side chains of lysozyme in crystal and solution simulations [12].

**Fig 1:**
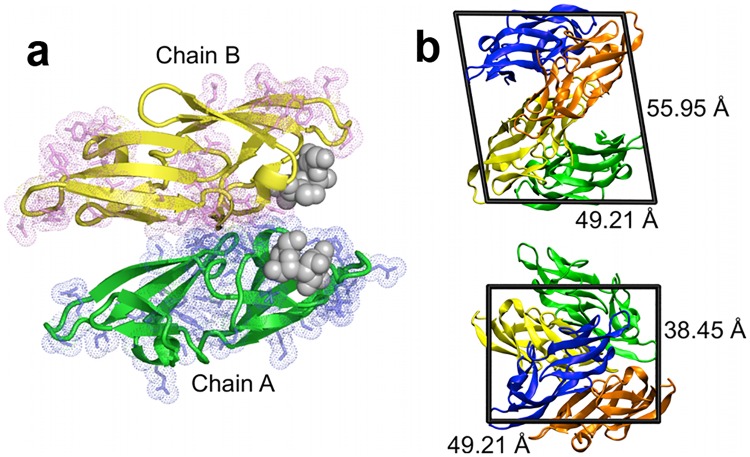
Crystal environment of CVN. (a) The two crystallographically independent molecules (A and B) of the P51G-m4-CVN complex (PDB ID: 2RDK) [17]. The secondary structure of molecules A and B are shown in green and yellow cartoon representation, and the di-mannose ligands are depicted as grey spheres. Residues participating in crystal contacts with neighboring molecules are shown as sticks and dots, colored in blue and magenta for molecules A and B, respectively. (b) The simulated triclinic CVN unit cell comprises four chains: two chain A’s (blue and green) and two chain B’s (orange and yellow). One A/B pair (e.g., the green and yellow chains) forms the asymmetric unit in the original monoclinic crystal structure.

Each of the aforementioned MD-based studies focused on a single model system to establish crystal simulation protocols and to quantitatively assess side-chain dynamics. In a similar vein, we consider Cyanovirin-N (CVN). While CVN has been largely studied for its microbicide potential [13–15], the P51G-m4-CVN mutant (Fig 1a) also presents a tractable model system to investigate the effect of the crystal environment on side-chain conformational dynamics: P51G-m4-CVN is relatively small and rigid (102 amino acids with two disulfide bonds), stays in monomeric form in solution (wild type CVN forms a domain-swapped dimer), represents a functionally relevant state (i.e., demonstrates activity against the oligomannose CVN substrate) [16] and has a high-resolution (1.35 Å) X-ray structure in complex with di-mannose available [17]. We previously investigated [18] the conformational dynamics of an arginine residue (Arg76) near the di-mannose biding site of P51G-m4-CVN that was proposed to play an important role in ligand binding [17]. Through crystal and solution MD simulation, we found that Arg76 was trapped in a single conformation in the crystal, which did not correspond to the dominant solution state. This observation brought into question the putative role of Arg76 in ligand interaction. It also prompted us to perform additional crystal and solution simulations of CVN in order to assess the effect of the crystal environment on conformation and dynamics across all of the side chains in the protein.

In this work, we assessed the effect of the crystal environment by quantitatively comparing side-chain conformational dynamics of the P51G-m4-CVN:di-mannose complex in solution and crystal MD simulations. Side-chain ensembles obtained from simulation show reasonable agreement with X-ray data, especially for residues participating in crystal contacts during crystal simulation. The simulations support the use of surface engineering to facilitate protein crystallization and provide insight into the influence of crystal packing on side chains in CVN. Combined with crystal simulation protocols, our quantitative analyses performed in this study demonstrate a practical framework for obtaining a broader view of crystal-packing effects on side-chain conformational dynamics by considering a larger set of proteins.

## Materials and Methods

### MD Simulation

A detailed description of the crystal and solution simulation setup for the P51G-m4-CVN:di-mannose complex (PDB ID: 2RDK) can be found in reference [18]. The crystal structure comprises two independent chains (A and B), both of which have two copies in the unit cell (i.e., four total chains: A1, A2, B1, and B2; Fig 1b). In short, all simulations were performed using the Amber10 package [19, 20]. The FF99SB [21, 22] and GLYCAM06 [23] force-field parameters were employed for the protein and ligand, respectively. Both solution and crystal simulations were performed with explicit water molecules. Crystal simulation does not have a bulk solvent region, which is required for solution simulation. Periodic boundary conditions for crystal simulation were set to coincide with the unit cell geometry. The Particle Mesh Ewald method [24] was used for calculating non-bonded interactions with a cutoff of 10 Å for direct calculations. An extensive equilibration phase was first performed for the crystal simulations to ensure an appropriate density of the system [18]. We performed production simulations in solution and in the crystal environment for a total of 32 ns each in the NPT and NVT ensembles, respectively. For the crystal simulations, the presence of multiple copies of proteins with identical packing environments in the unit cell (two chain A’s and two chain B’s) results in increased sampling (128 ns total– 64 ns for both chains A and B). The temperature was maintained at 300 K (which closely corresponds to the crystal growth temperature of 298 K) [17]. All trajectories were processed with ptraj [20] and visualized with VMD [25].

### Side-Chain Conformations

Side-chain conformations were defined by rotameric states, as listed in the Penultimate Rotamer Library [26]. We measured side-chain torsion angles sampled during simulation using an in-house edited version of the *rotamer* program that is part of CCP4 [27]. For each residue, the torsion angles were mapped to the rotamer library to identify the nearest rotameric state. The rotameric state IDs used in the following data correspond to the rotamer tables given in http://www.ccp4.ac.uk/html/rotamer_table.html. Rotamers for each residue can be viewed and downloaded at http://kinemage.biochem.duke.edu/databases/rotkins.php [26]. Conformations were defined for all rotameric residues (i.e., not Ala and Gly) for snapshots extracted every 10 ps over the last half of the solution and the crystal simulations. Disulfide-bonded cysteins were also excluded from analysis. We constructed normalized rotamer probability distributions for the analyses described below. In the case of the crystal simulations, rotameric data was combined for the identical chains in the unit cell (A1/A2 and B1/B2). In the P51G-m4-CVN crystal structure, 81/101 residues in chain A and 80/100 residues in chain B have rotamers.

### Definition of Crystal Contacts

We defined a residue as participating in crystal packing if it has any heavy atom within 4 Å of a heavy atom belonging to a crystal neighbor. Such intermolecular pairs were identified with the *ncont* program that is part of CCP4 [27]. Under this criterion, chains A and B in CVN have 41 and 40 residues participating in a crystal contact, respectively. Thirty-six of the contacting residues in each chain have rotamers.

### Solvent Accessibility

To determine if a given residue in CVN is solvent-exposed, we computed its relative solvent accessibility as the per-residue solvent accessible surface area (SASA) divided by the theoretical maximum accessibility of the residue in an Ala-X-Ala peptide [28]. The SASA per residue was calculated separately for chains A and B using the AREAIMOL program [28, 29] in CCP4 [27] with a probe sphere radius of 1.4 Å and a density of 15 points per Å^2^. A residue was defined as exposed if its relative accessibility was > 0.2 [2].

### Comparison of Side-Chain Conformational Ensembles

**Fig 2:**
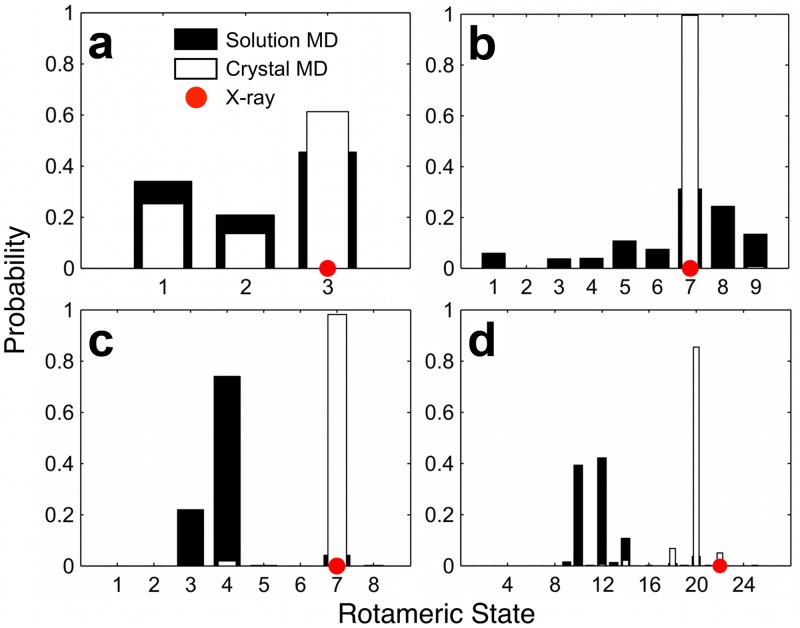
Representative rotamer probability distributions for several residues in CVN. Rotameric states are denoted by numbers on the horizontal axis, and correspond to the order in which they appear in the Penultimate Rotamer Library [26] for each residue. (a) Leu69 in chain B (B:Leu69) does not participate in a crystal contact and exhibits high conformational overlap between the distributions obtained from solution (black bars) and crystal (white bars) MD simulation (*OC* = 0.83), roughly no change in conformational entropy (*T*Δ*S*_conf_ = –0.10 kcal/mol), and agreement between the rotamer observed in the X-ray structure (denoted by the red dot) and the most dominant rotamer sampled in both simulations. (b) A:Gln6 participates in a crystal contact and shows an *OC* = 0.32 and *T*Δ*S*_conf_ = –1.06 kcal/mol, while the dominant rotamer agrees among the X-ray data and both simulations. (c) A:Glu56 is contacting and exhibits an *OC* = 0.06 and *T*Δ*S*_conf_ = –0.36 kcal/mol, and the X-ray conformation agrees with crystal MD. (d) B:Lys74 is non-contacting and displays an *OC* = 0.08 and *T*Δ*S*_conf_ = –0.44 kcal/mol, and the dominant rotameric states and X-ray conformation disagree.

Rotamer probability distributions were computed for each rotameric residue (S1–S14 Figs). These distributions were used for several quantitative comparisons of side-chain conformational dynamics. Several representative histograms and their corresponding measures of conformational dynamics, as described below, are presented in Fig 2. For crystal simulation, the original 2RDK crystal structure [17] in space group P2(1) with two crystallographically independent molecules (A and B) in the asymmetric unit was converted to a triclinic one (space group P1) with the same unit cell parameters but four symmetrically independent molecules. Despite the formal loss of the 2(1) symmetry, these four molecules can be split into two pairs of protein chains (A1/A2 and B1/B2). For a given pair (A1/A2 or B1/B2), the molecules reside in nearly identical crystal environments. Therefore, the rotamer probability histograms in molecule A1 were combined with A2, and the same was done for molecules B1 and B2. The use of crystal simulations with multiple molecules in the crystal lattice was proposed as a method to accelerate conformational sampling and is described in detail elsewhere [30]. Since molecules A and B are not crystallographically symmetric, they are in different crystal environments. Thus, the conformational sampling of side chains in these two molecules is expected to be different even for solvent-exposed residues, and we consider side-chain conformational space in molecules A and B separately. Finally, for the purpose of this study, we consider the dynamics of solvent-exposed residues in CVN. We divide these residues into two groups: those that interact with neighboring molecules in the crystal lattice (“contacting”) and those that do not (“non-contacting”) (see Definition of Crystal Contact above for details). The analyses presented below were performed for all solvent-exposed residues, as well as for the subsets of contacting and non-contacting solvent-exposed side chains.

#### Likelihood Score

We calculated a likelihood score to evaluate how well the rotamer probability distributions from the solution and crystal simulations reflect the X-ray side-chain conformations. From simulation, we obtain the probability *P*_*i*_^*y*^(*x*) that a given residue *i* is in rotameric state *x* under condition *y* (solution or crystal). For each residue, the rotamer observed in the X-ray data is denoted as *x*_*i*_^*xray*^. As a reference, we consider the null model *P*_*i*_^*rand*^(*x*) = 1/*N*, which is the probability that residue *i* is in state *x* if all rotamers *N* defined for this residue were equally accessible. We then calculate the average of a relative likelihood score as

$$L_{y}=\frac{1}{n_{res}}\sum_{i=1}^{n_{res}}\frac{P_{i}^{y}(x_{i}^{xray})}{P_{i}^{rand}(x_{i}^{xray})},$$ (1)

where *n*_*res*_ is the number of rotameric residues considered. Normalizing by *P*_*i*_^*rand*^(*x*) ensures that residues with a small number of accessible rotamers, which have a higher probability to match the experimental rotamer at random, do not dominate the score. We also report the quotient *L*_*crystal*_*/L*_*solution*_: values greater than 1 indicate that the crystal simulations show a higher correspondence than do the solution simulations with the X-ray data and vice versa.

#### Rotamer Agreement

Side-chain conformations in simulation and in the X-ray model were considered in agreement if one of the two most dominant rotamers (the two histogram bins with the greatest number of counts) visited during simulation was the same as that observed in the experimental structure. Rotameric states were determined to be the same if each side-chain torsion angle was within a ± 30° tolerance of the corresponding χ angle listed in the rotamer library. We note that this criterion is stringent for longer residues (i.e., those with more than two torsion angles along the side chain), as each angle must match to count as conformational agreement.

#### Overlap Coefficient

To assess the agreement between rotamer probability distributions for solvent-exposed residues in solution and crystal simulation, we compute an overlap coefficient (*OC*) [31, 32]:

$$OC=\sum_{i=1}^{N}\text{min}\left(p_{i}^{S},p_{i}^{C}\right).$$ (2)

*p*^*S*^ and *p*^*C*^ are the probabilities for rotamer state *i* in solution and crystal simulation, respectively. The sum is over all rotameric states *N* available to a particular residue. *OC* values range from 0 to 1, representing zero and full histogram overlap, respectively. Analyzing conformational agreement with the Kullback-Leibler divergence (a score for which a value of zero indicates perfect histogram overlap) [33] yields the same trend as the *OC* values (correlation of –0.9). Per residue *OC* values computed for two equal-length and non-overlapping trajectory segments show an R-value of 0.76, indicating sufficient sampling. We used per-residue *OC* values from these two trajectory segments as an estimate of uncertainty (S15a and S15b Fig).

#### Conformational Entropy

To compare side-chain dynamics in the crystal and solution simulations, we analyze the conformational entropy of side chains on the protein surface. This conformational entropy can be expressed as a multidimensional well for each side chain [34, 35]:

$$S_{conf}=\sum_{i=1}^{N}p_{i}S_{i}^{therm}-k_{B}\sum_{i=1}^{N}p_{i}\text{ln}p_{i},$$ (3)

where the index *i* runs over all *N* rotameric states for a particular residue and *p*_*i*_ is the probability of the *i*^*th*^ rotameric state. *S*_*conf*_ is split into two terms: the first term corresponds to the thermal motion within a given conformational well ($S_{i}^{therm}$) and the second term represents the configurational entropy arising from the sampling of distinct rotameric states. In this approximation, side-chain movement of each residue between these states is considered to be independent from its neighbors, which may result in the overestimation of the total entropy, but allows for a more computationally efficient calculation. We further simplify the analysis with the assumption that $S_{i}^{therm}$ is the same in each rotameric state for a particular side chain. In the current study, we are interested in Δ*S*_*conf*_ for the same residue in different environments (solution versus crystal) at the same temperature, and thus the thermal motion terms cancel each other [35]. Thus, the conformational entropy per side chain reduces to only the configurational (second) term in Eq 3. *S*_*conf*_ is also calculated with just the configurational component in several studies focusing on protein folding [36–41]. We define Δ*S*_conf_ as *S*_conf,crystal_ − *S*_conf,solution_, and these entropy values are reported as *T*Δ*S*_conf_ (*T* = 300 K). Per residue *T*Δ*S*_conf_ values determined for two equal-length and non-overlapping trajectory segments yield an R-value of 0.54, suggesting adequate sampling. In the same manner as the *OC* above, we considered the results from these two trajectory segments to estimate the per-residue uncertainty in *T*Δ*S*_conf_ (S15c and S15d Fig).

## Results

### Comparison to X-ray Structure Data

We first compared the experimental side-chain conformations in the X-ray data to the rotamers sampled during solution MD simulation. We calculated a likelihood ratio (see Materials and Methods) that assesses the degree to which our simulations capture side-chain conformations observed in the X-ray structure relative to a null model. In the null model, each rotamer for a given residue is considered to be equally accessible. With this approach, the significance of agreement is appropriately considered for residues with different sizes and thus number of rotameric states.

The likelihood scores averaged across solvent-exposed residues in CVN are all well above unity (left-hand side and middle of Table 1), which indicates better agreement with experiment than simply matching rotamers at random. For the set of all solvent-exposed residues, the rotamers in solution and crystal simulation are ~2.5–2.9 and ~3.4–4.0 times, respectively, more likely to match the X-ray data than the null model. These scores are roughly equivalent to agreement percentages of ~50–60% and ~70–80% for the solution and crystal simulations, respectively, assuming that the surface residues have five rotameric states on average. The ratio reaches as high as ~5 for contacting residues in the crystal simulations. Differences in the likelihood ratios between the sets of contacting and non-contacting residues could be due to differences in residue composition (S16 Fig). For example, while roughly a third of the residues in both of these sets in chain A correspond to longer and more flexible residues (ARG-GLN in S16 Fig), nearly half (0.44) and just a quarter (0.27) of the residues in the contacting and non-contacting sets, respectively, represent shorter and less flexible side-chains (TYR-SER). A similar trend is observed in chain B. We also calculated that ~60% and ~75% of the side-chain conformations in the experimental structure correspond to one of the two most populated rotameric states from solution and crystal MD simulation, respectively (S1 Table). In the crystal simulations, this level of agreement with experiment increases to 84% for the set of contacting residues. A caveat of such an approach is that the comparison is biased toward smaller residues that have fewer accessible rotamers (e.g., Thr with three states) and are thus more likely to match with experiment at random. A certain level of disagreement should be expected from these analyses due to the fact that the data for the X-ray structure were collected 100 K while our simulations were performed at room temperature (see Discussion for more details).

**Table 1:** Likelihood ratios for sampling X-ray rotamers in solution and crystal simulation^a^.

	Solution Simulation			Crystal Simulation			Crystal/Solution		
chain^b^	all^c^	cont	non-cont	all	cont	non-cont	all	cont	non-cont
A	2.49	3.10	1.60	3.42	4.48	1.87	1.37	1.45	1.16
B	2.89	3.09	2.63	3.97	4.93	2.74	1.38	1.60	1.04

^a^See the “Likelihood score” subsection in Methods.

^b^In chains A/B, at total of 54/55 are located on the protein surface, of which 32/31 are contacting and 22/24 are non-contacting.

^c^Values are averaged over the set of all solvent-exposed rotameric residues (“all”) and for the subsets of contacting (“cont”) and non-contacting (“non-cont”) solvent-exposed residues. For residues with alternate conformations in the crystal structure, conformation A was used.

Overall, the likelihood scores and the comparison of the most dominant rotameric states indicate that the side-chain conformational space sampled during our MD simulations agrees well with experiment. Thus, our further comparative analysis of side-chain ensembles in solution and in the crystal focuses on results obtained from the MD simulations.

### Comparison of Side-Chain Conformational Dynamics in Crystal and Solution MD

We first assessed the relative performance of solution and crystal simulation to reproduce X-ray side-chain conformations by taking the quotient of likelihood ratios (see Methods and the right-hand side of Table 1). For this quotient, values greater than 1 indicate that the crystal simulations exhibit a higher level of agreement with X-ray side chains than the solution simulation. Values less than 1 signify the opposite trend. For the set of all solvent-exposed rotameric residues, the ratio is greater than 1 (~1.4) for both chains A and B (Table 1). The ratio increases to 1.45 and 1.60 for the set of residues that participate in crystal contacts, and diminishes closer to 1 for the group of non-contacting residues. Thus, this analysis indicates that the crystal simulations show a higher degree of correspondence than do the solution simulations with the X-ray data.

Rotamer probability histograms obtained from solution and crystal MD simulations are shown in Fig 2 and in S1–S14 Figs. To compare side-chain conformations in these ensembles, we computed the overlap coefficient (*OC*; Eq 2) between the probability distributions from the solution and crystal MD simulations. *OC* values closer to one and zero correspond to better and poorer agreement between histograms, respectively. As shown on the left-hand side of Table 2, the average *OC* (<*OC*>) across all solvent-exposed residues is ~0.6–0.7, indicating that the majority of the side chains sample a comparable region of conformational space in solution and in the crystal. <*OC*> for the non-contacting set is higher than that of the contacting set by 0.05 (chain A) and 0.15 (chain B).

**Table 2:** Average histogram overlap and entropy change in crystal and solution MD for solvent-exposed residues in CVN^a^.

	<*OC*>			<*T*Δ*S*_conf_>		
chain	all	cont	non-cont	all	cont	non-cont
A	0.70 (0.28)	0.68 (0.30)	0.73 (0.25)	−0.14 (0.28)	−0.20 (0.33)	−0.05 (0.15)
B	0.63 (0.31)	0.57 (0.34)	0.72 (0.24)	−0.10 (0.24)	−0.14 (0.27)	−0.05 (0.21)

^a^<*OC*> and <*T*Δ*S*_conf_> (kcal/mol) are the average values over the set of all solvent-exposed rotameric residues (“all”) and for the subsets of contacting (“cont”) and non-contacting (“non-cont”) residues. The standard deviation is reported in parentheses next to the average.

**Fig 3:**
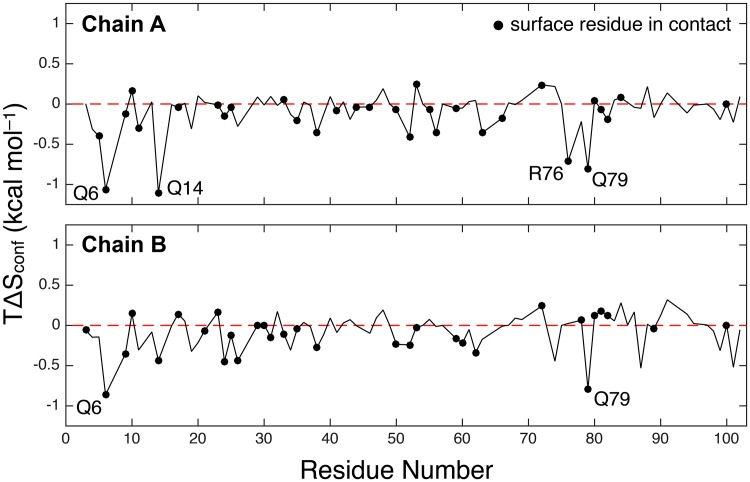
Differences in side-chain conformational entropy in the crystal for CVN. *T*Δ*S*_conf_ is shown as a function of residue number for chains A (top) and B (bottom). Negative *T*Δ*S*_conf_ values denote a loss in side-chain conformational entropy in the crystal; a dashed horizontal line is shown in red at *T*Δ*S*_conf_ = 0 (i.e., no change in side-chain dynamics). Residues that exhibit a large decrease in *T*Δ*S*_conf_ are denoted by their one-letter amino acid abbreviation and number. Contacting surface residues are indicated by filled circles.

The rotamer histograms were also used to calculate the average change in residue conformational entropy (<*T*Δ*S*_conf_ >) as a measure of side-chain dynamics (right-hand side of Table 2). We compare side-chain entropies in the crystal and in solution by only considering the configurational component of the conformational entropy, which is a reasonable approximation [35] (see Materials and Methods for more detail). Δ*S*_conf_ is defined as *S*_conf,crystal_−*S*_conf,solution_, and thus values less than zero represent a decrease in side-chain dynamics in the crystal compared to in solution. A negative <*T*Δ*S*_conf_ > is observed for all solvent-exposed residues as well as for the contacting and non-contacting subsets in both chains A and B. This effect is four (chain A) and three (chain B) times greater in magnitude for the contacting set compared to the non-contacting set. While the values of <*T*Δ*S*_conf_ > are modest, a significant loss in conformational entropy is observed for several long, polar and contacting surface residues (*T*Δ*S*_*conf*_ approaches –1 kcal/mol; Fig 3). No residue exhibits a *T*Δ*S*_conf_ greater than ~0.3 kcal/mol. The per-residue conformational dynamics in chains A and B is expected to be different for solvent-exposed residues in CVN, as the two chains reside in different crystal packing environments.

**Fig 4:**
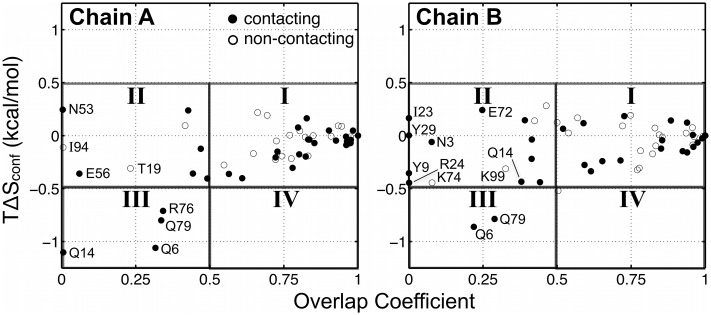
Comparison of changes in side-chain conformation (*OC*) and dynamics (*T*Δ*S*_conf_). Data is shown for all rotameric residues in CVN chains A (left) and B (right). The plots are divided into four quadrants, which reflect the effect of the crystal environment on conformation and dynamics: I) small or no effect on conformation and dynamics; II) change in conformation, small effect on dynamics; III) change in conformation and in dynamics; and IV) change in dynamics, small effect on conformation. Filled data points denote residues participating in a crystal contact, and open circles represent non-contacting residues.

**Fig 5:**
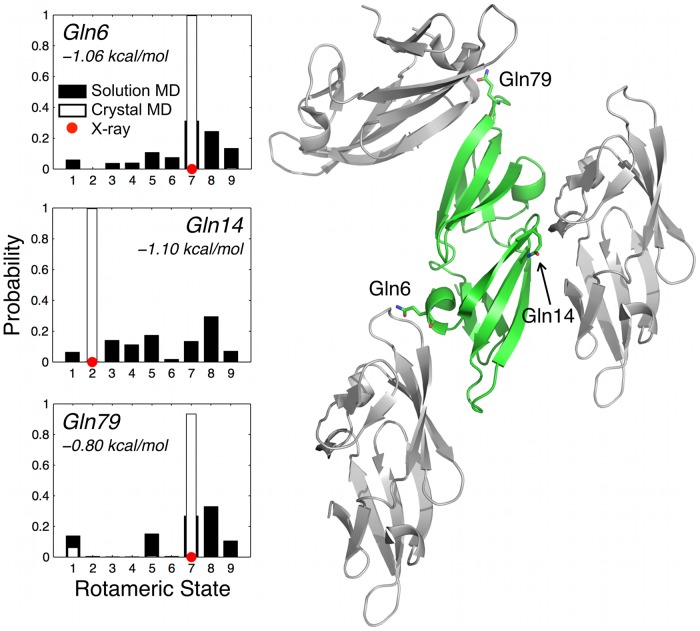
Crystal packing notably diminishes conformational dynamics of several solvent-exposed glutamine residues in CVN. The histograms at left show that three glutamines in chain A (Gln6, Gln14, and Gln79) are trapped in their respective X-ray conformations (red circles on the histograms) during crystal MD simulation (white bars), while the residues sample several different rotamers in solution simulation (black bars). *T*Δ*S*_conf_ values are listed under the residue name/number in each histogram. Crystal contacts between these three glutamines and neighboring chains in the crystal are shown at right.

We glean further insight into the effect of the crystal on side-chain conformational dynamics by examining the relationship between *OC* and *T*Δ*S*_conf_ values for each residue (Fig 4). The plots are divided into quadrants to emphasize the relationship between side-chain conformation and dynamics. Filled and open circles denote crystal-contacting and non-contacting residues, respectively. Region I comprises residues for which the crystal has little or no effect on conformation or dynamics, and is bounded by *OC* values of 0.5 to 1 and *T*Δ*S*_conf_ values of –0.5 to +0.5 kcal/mol. (These boundaries correspond to approximately twice the standard deviation of the <*OC*> and <*T*Δ*S*_conf_ > values reported in Table 2.) The majority of residues fall within this region. The residues for which conformation and/or dynamics are notably different in solution and in the crystal are indicated by data points located outside of region I. Most of the affected side chains undergo a change in conformation (*OC* < 0.5) with little or no change in dynamics (Fig 4, region II). Both contacting and non-contacting residues fall into this scenario. For the long, polar contacting residues highlighted in Fig 3, the strong decrease in dynamics is accompanied by a change in conformation (Fig 4, region III). Interestingly, we do not observe the scenario in which dynamics is notably decreased while maintaining a dominant solution conformation (Fig 4 region IV). In other words, if the change in dynamics is noticeable for some residues (for example, as indicated by the thermal factor), then its conformation is also most likely affected. Rotamer histograms and crystal packing interactions of several of the most affected residues are highlighted in Fig 5.

## Discussion

X-ray models play an essential role in functional interpretations of proteins. We must carefully evaluate the structural features of these models in the context of the crystal environment. Toward this aim, we have compared side-chain conformational dynamics in solution and crystal MD simulations of a protein for which a high-resolution X-ray structure is available.

Side-chain conformational ensembles obtained from the simulations for solvent-exposed residues in CVN exhibit a high level of correspondence with the X-ray model, as revealed through the likelihood analysis. Although differences in side-chain conformations in simulation and in the crystal structure may, at least to some degree, be due to a rigorous definition of conformational similarity (i.e., all torsion angles along the side chain must match), other factors may contribute. Firstly, CVN was simulated near the crystal growth temperature of 298 K instead of at the cryogenic temperature for data collection (100 K), as low temperatures may be unsuitable for standard MD force fields. Moreover, cryocooling increases the extent of lattice contacts, especially for longer residues (Gln, Glu, Arg, and Lys) [42], and may remodel over a third of all side chains relative to structures solved at room temperature or even eliminate conformations essential for function [8]. Taking into account these considerations, it is possible that the simulated side-chain ensembles more closely represent local conformational dynamics in the crystal before flash freezing, which could account for a certain level of disagreement between MD and X-ray. Accordingly, the deposition of an ensemble of X-ray models [43], for which precedent exists from NMR, may lead to a more comprehensive comparison of side-chain conformational dynamics in the solution and crystal environments.

While in simulation the average loss in side-chain conformational entropy in the crystal is minimal (even for contacting residues), significant decreases in entropy occur for a handful of long and polar contacting residues on the protein surface. *T*Δ*S*_conf_ for these cases approaches –1 kcal/mol. This value is comparable to the average loss in conformational entropy per residue upon protein folding (–0.95 kcal/mol) [44], indicating that crystal packing can significantly diminish conformational dynamics for long, solvent-exposed side chains. To fully assess the free energy contribution to packing interface energetics, both entropy and enthalpy terms need to be estimated, as they typically compensate each other in a complex manner [45]. While our finding supports application of the SER method [5, 6] in which longer residues on the protein surface are replaced with shorter ones (e.g. Ala) to facilitate crystallization by minimizing losses in conformational entropy, one must also consider that this method may affect favorable enthalphic interactions. Nevertheless, our study provides detailed pictures of changes in side-chain conformational dynamics and entropy upon crystal packing formation.

Finally, our results suggest a model for the effect of crystallization on side-chain conformational dynamics. All residues that exhibit different behavior in crystal versus solution MD simulation show a notable change in conformation and/or dynamics. The large majority of residues that exhibit a change in conformation still retains a similar degree of dynamics in the crystal (region II in Fig 4). These cases result from the shift in either broadly distributed or in rather restricted rotamer populations (S17 Fig), which selects a minor solution state or a new rotamer. Moreover, several of these cases correspond to non-contacting residues, indicating that longer-range effects (e.g., electrostatics and solvation), in addition to direct lattice contacts, may play a role in influencing side-chain conformation [2]. Such conformational changes can also be accompanied by a strong decrease in dynamics for long, polar and contacting residues on the protein surface (region III in Fig 4), thus counteracting crystal formation. Inspection of rotamer state histograms shows that, for some residues, crystal packing may perturb side-chain conformations to rotamer states that are already observed in the solution environment and correspond to either major or minor populations. Such a scenario would correspond to a conformational selection model [46]. For some residues, new rotamer states are realized as the result of packing, which can be characterized by an induced fit model [47]. Both induced fit and conformational selection have been proposed in studies of the influence of the crystal environment on global protein structure [48–51]. The possibility of multiple ways through which crystal packing affects protein conformational dynamics underscores the complexity of the crystal environment. The methods for crystal MD simulation and the quantitative analyses demonstrated in this study can be used to investigate a larger set of X-ray structures in order to more fully understand this effect.

## Supporting Information

S1 Fig Rotamer probability distributions for rotameric residues in Cyanovirin-N: chain A, residues 3–18. The title of each individual histogram specifies the chain and the residue three-letter abbreviation followed by the residue number, and is denoted in red font if the residue participates in a crystal contact. Rotameric states are indicated by numbers on the horizontal axis, and correspond to the order in which they appear in the Penultimate Rotamer Library for each residue (see ref. [26] in the main text). Black and white bars correspond to distributions obtained from solution and crystal MD, respectively. The red circles on the horizontal axis denote the rotamer observed in the X-ray structure; red crosshairs indicate alternate X-ray conformations. These details are the same for S1–S14 Figs.
(TIFF)

S2 Fig Rotamer probability distributions for rotameric residues in Cyanovirin-N: chain A, residues 19–33. For details, see caption to S1 Fig.
(TIFF)

S3 Fig Rotamer probability distributions for rotameric residues in Cyanovirin-N: chain A, residues 34–46. For details, see caption to S1 Fig.
(TIFF)

S4 Fig Rotamer probability distributions for rotameric residues in Cyanovirin-N: chain A, residues 47–60. For details, see caption to S1 Fig.
(TIFF)

S5 Fig Rotamer probability distributions for rotameric residues in Cyanovirin-N: chain A, residues 61–78. For details, see caption to S1 Fig.
(TIFF)

S6 Fig Rotamer probability distributions for rotameric residues in Cyanovirin-N: chain A, residues 79–90. For details, see caption to S1 Fig.
(TIFF)

S7 Fig Rotamer probability distributions for rotameric residues in Cyanovirin-N: chain A, residues 91–102. For details, see caption to S1 Fig.
(TIFF)

S8 Fig Rotamer probability distributions for rotameric residues in Cyanovirin-N: chain B, residues 3–18. For details, see caption to S1 Fig.
(TIFF)

S9 Fig Rotamer probability distributions for rotameric residues in Cyanovirin-N: chain B, residues 19–33. For details, see caption to S1 Fig.
(TIFF)

S10 Fig Rotamer probability distributions for rotameric residues in Cyanovirin-N: chain B, residues 34–46. For details, see caption to S1 Fig.
(TIFF)

S11 Fig Rotamer probability distributions for rotameric residues in Cyanovirin-N: chain B, residues 47–60. For details, see caption to S1 Fig.
(TIFF)

S12 Fig Rotamer probability distributions for rotameric residues in Cyanovirin-N: chain B, residues 61–78. For details, see caption to S1 Fig.
(TIFF)

S13 Fig Rotamer probability distributions for rotameric residues in Cyanovirin-N: chain B, residues 79–90. For details, see caption to S1 Fig.
(TIFF)

S14 Fig Rotamer probability distributions for rotameric residues in Cyanovirin-N: chain B, residues 91–101. For details, see caption to S1 Fig.
(TIFF)

S15 Fig Uncertainty estimation in the quantitative measures of side-chain conformational dynamics. *OC* (*a* and *b*) and *T*Δ*S*_conf_ (*c* and *d*) calculated over the last half of simulation (16–32 ns; solid line) for chains A (top panels) and B (bottom panels). Error bars represent the uncertainty in *OC* and *T*Δ*S*_conf_ for each residue. Measurements of these quantities were calculated during two equal-length, non-overlapping trajectory segments (16–24 ns and 24–32 ns) and then taken as lower and upper bound estimates. The error bars represent the interval encompassed by these bounds.
(TIF)

S16 Fig Residue composition of contacting and non-contacting solvent-exposed residues in CVN. Frequency of residues in the contacting (top panels) and non-contacting (bottom panels) sets of residues in CVN chains A (at left) and B (at right). Residues on the horizontal axis are ordered based upon the number of rotameric states: left (more rotamers) to right (less rotamers) according to the Penultimate Rotamer Library [26]. Ala and Gly do not have any rotamers, and Cys is excluded from the analysis since the cysteins in CVN participate in disulfide bonds.
(TIFF)

S17 Fig Rotamer probability distributions for several residues in CVN that show a change in conformation but not in dynamics. In the same manner as Fig 2 of the main text, rotameric states are denoted by numbers on the horizontal axis, and correspond to the order in which they appear in the Penultimate Rotamer Library [26] for each residue. Distributions obtained from solution and crystal simulation are shown with black and white bars, respectively, and the rotatmer observed in the X-ray structure is denoted by the red dot on the horizontal axis (legend in panel B). (a) Glu56 in chain A (A:Glu56, same as Fig 2C; *OC* = 0.06 and *T*Δ*S*_conf_ = –0.36 kcal/mol) is contacting, (b) A:Ile94 (*OC* = 0.01 and *T*Δ*S*_conf_ = –0.11 kcal/mol) is non-contacting, (c) B:Asn3 (*OC* = 0.08 and *T*Δ*S*_conf_ = –0.06 kcal/mol) is contacting, and (d) B:Gln14 (*OC* = 0.38 and *T*Δ*S*_conf_ = –0.44 kcal/mol) is contacting.
(TIF)

S1 Table Agreement between dominant rotameric states from MD and X-ray for solvent-exposed residues in CVN. Side chains in simulation and experiment are determined to be in agreement if one of the two most dominant rotamers from simulation matches the X-ray conformation. Percent agreement is averaged over all solvent-exposed rotameric residues (“all”) and for the subsets of contacting (“cont”) and non-contacting (“non-cont”) solvent-exposed residues. For residues with alternate conformations in the crystal structure, conformation A was used for comparison.
(DOCX)
