# Supplementary figures and images for: Effect of the Crystal Environment on Side-Chain Conformational Dynamics in Cyanovirin-N Investigated through Crystal and Solution Molecular Dynamics Simulations
Source: PLoS One. 2017 Jan 20;12(1):e0170337. doi: 10.1371/journal.pone.0170337 (PMC5249168; doi:10.1371/journal.pone.0170337)

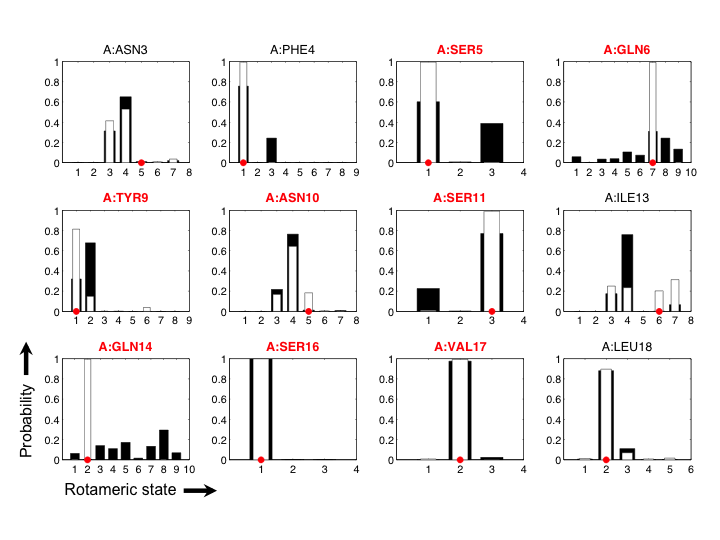

Supplement: S1 Fig — The title of each individual histogram specifies the chain and the residue three-letter abbreviation followed by the residue number, and is denoted in red font if the residue participates in a crystal contact. Rotameric states are indicated by numbers on the horizontal axis, and correspond to the order in which they appear in the Penultimate Rotamer Library for each residue (see ref. [26] in the main text). Black and white bars correspond to distributions obtained from solution and crystal MD, respectively. The red circles on the horizontal axis denote the rotamer observed in the X-ray structure; red crosshairs indicate alternate X-ray conformations. These details are the same for S1–S14 Figs. (TIFF) [file pone.0170337.s001.tiff]

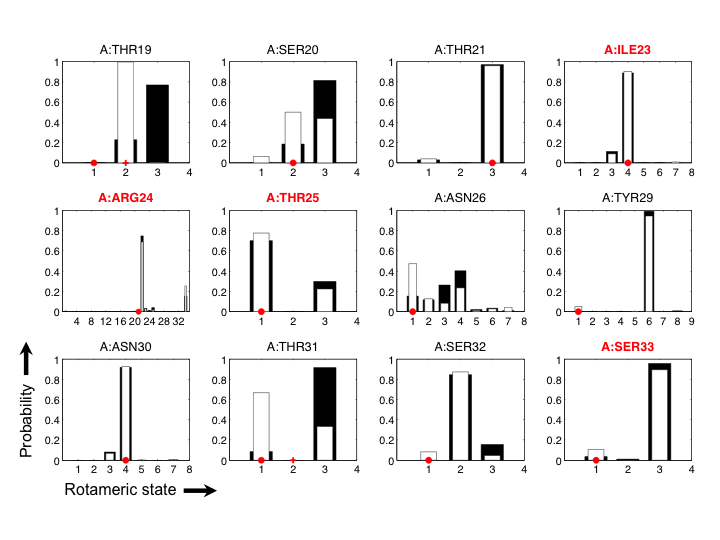

Supplement: S2 Fig — For details, see caption to S1 Fig. (TIFF) [file pone.0170337.s002.tiff]

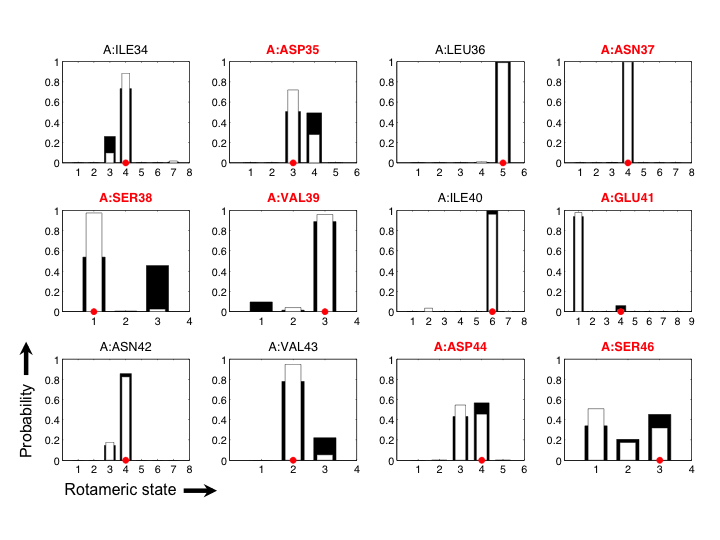

Supplement: S3 Fig — For details, see caption to S1 Fig. (TIFF) [file pone.0170337.s003.tiff]

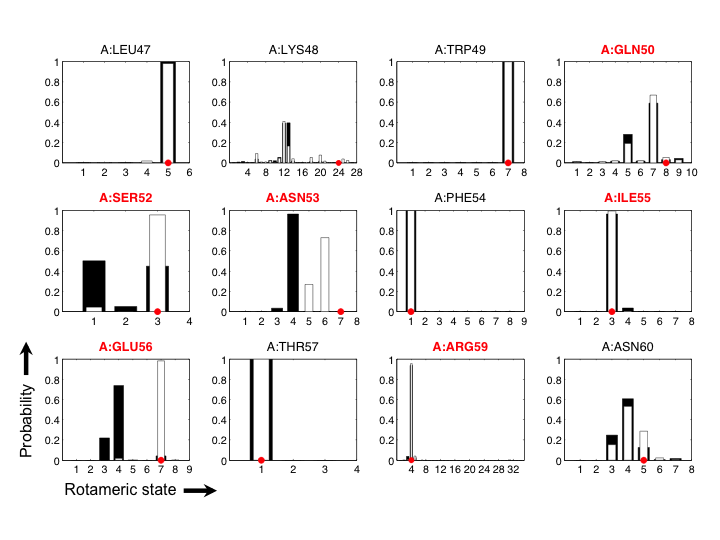

Supplement: S4 Fig — For details, see caption to S1 Fig. (TIFF) [file pone.0170337.s004.tiff]

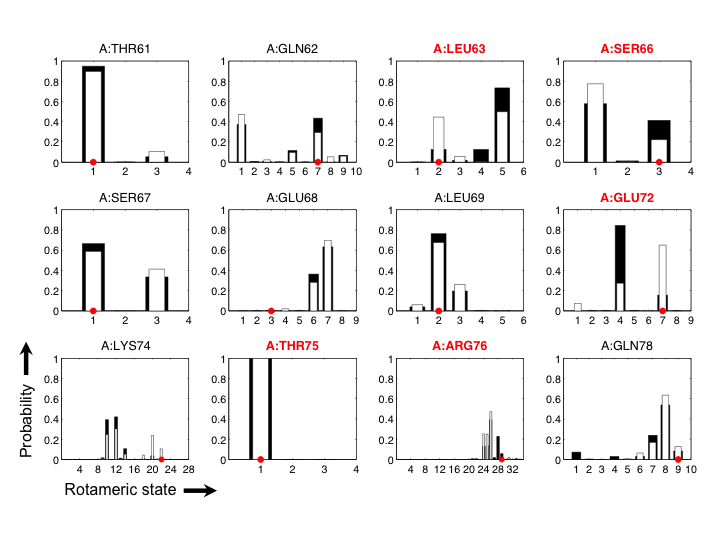

Supplement: S5 Fig — For details, see caption to S1 Fig. (TIFF) [file pone.0170337.s005.tiff]

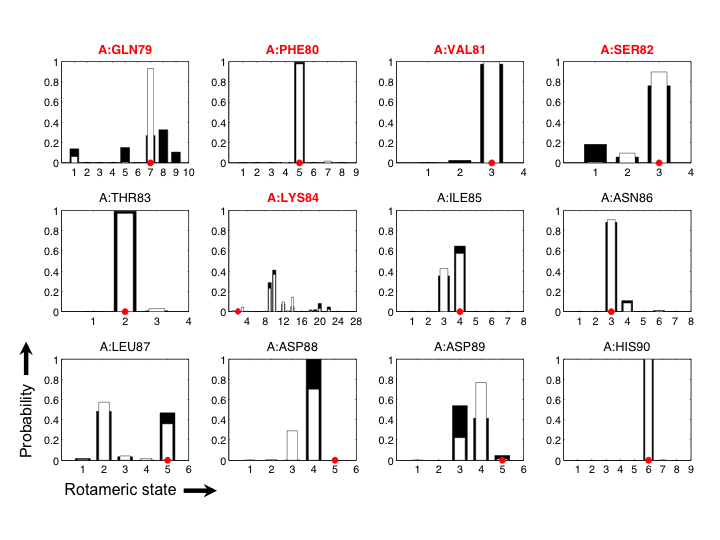

Supplement: S6 Fig — For details, see caption to S1 Fig. (TIFF) [file pone.0170337.s006.tiff]

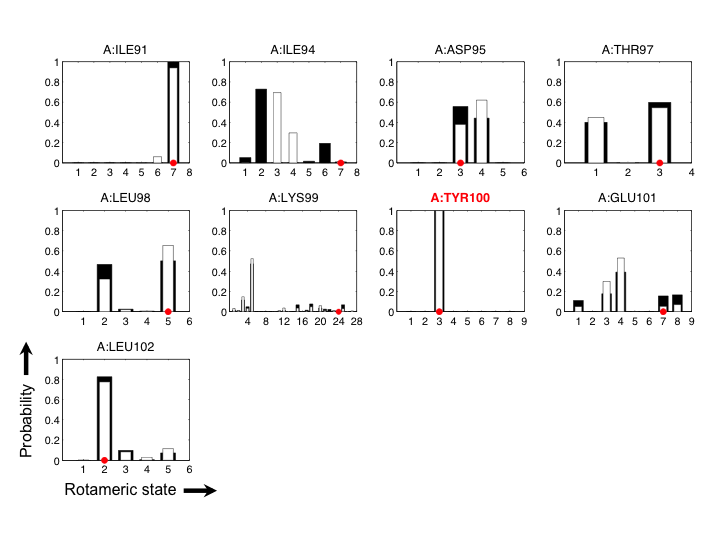

Supplement: S7 Fig — For details, see caption to S1 Fig. (TIFF) [file pone.0170337.s007.tiff]

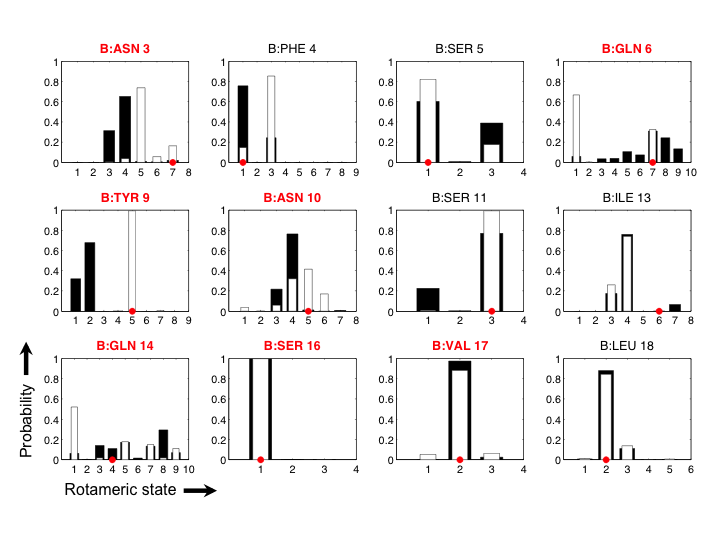

Supplement: S8 Fig — For details, see caption to S1 Fig. (TIFF) [file pone.0170337.s008.tiff]

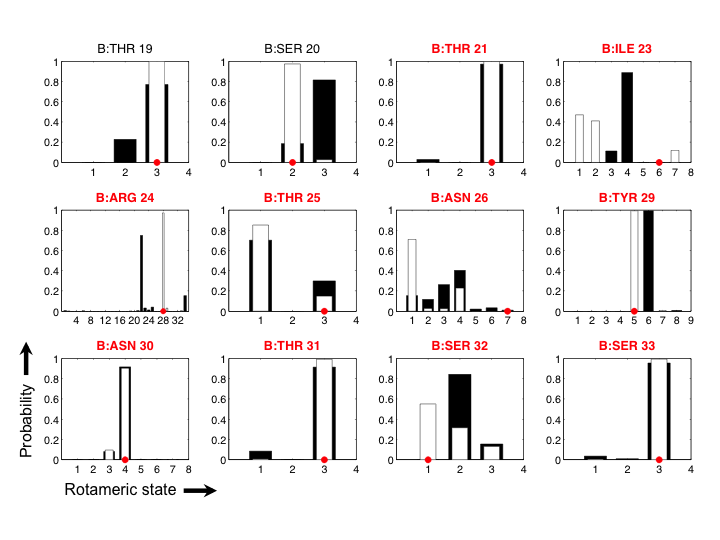

Supplement: S9 Fig — For details, see caption to S1 Fig. (TIFF) [file pone.0170337.s009.tiff]

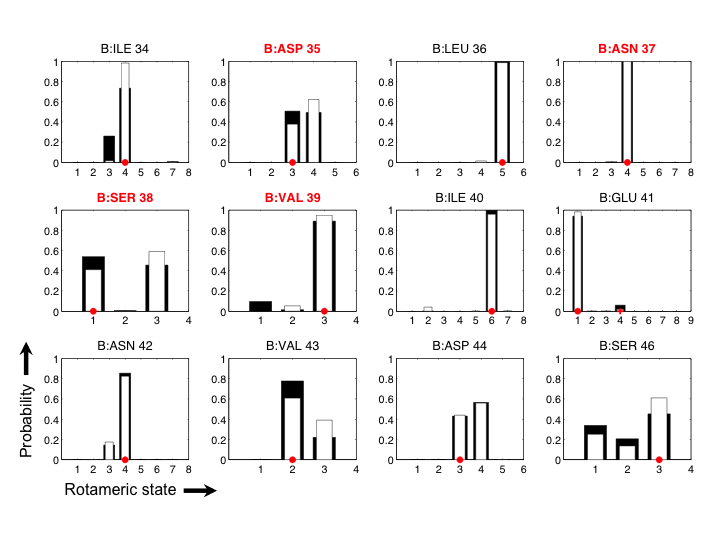

Supplement: S10 Fig — For details, see caption to S1 Fig. (TIFF) [file pone.0170337.s010.tiff]

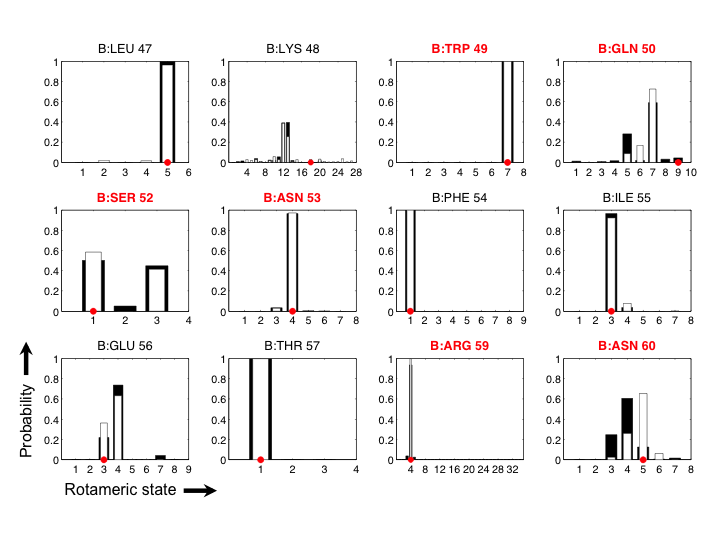

Supplement: S11 Fig — For details, see caption to S1 Fig. (TIFF) [file pone.0170337.s011.tiff]

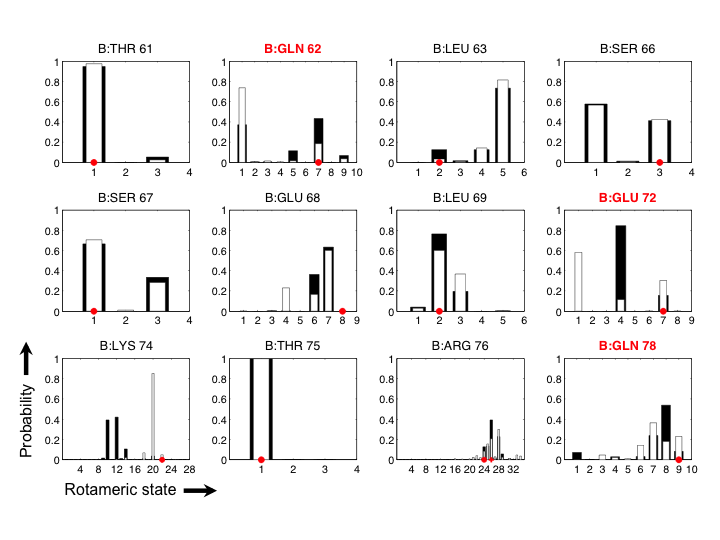

Supplement: S12 Fig — For details, see caption to S1 Fig. (TIFF) [file pone.0170337.s012.tiff]

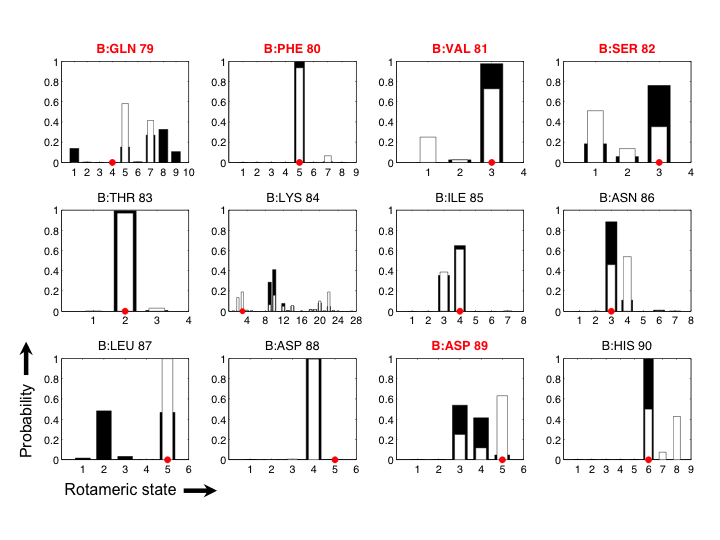

Supplement: S13 Fig — For details, see caption to S1 Fig. (TIFF) [file pone.0170337.s013.tiff]

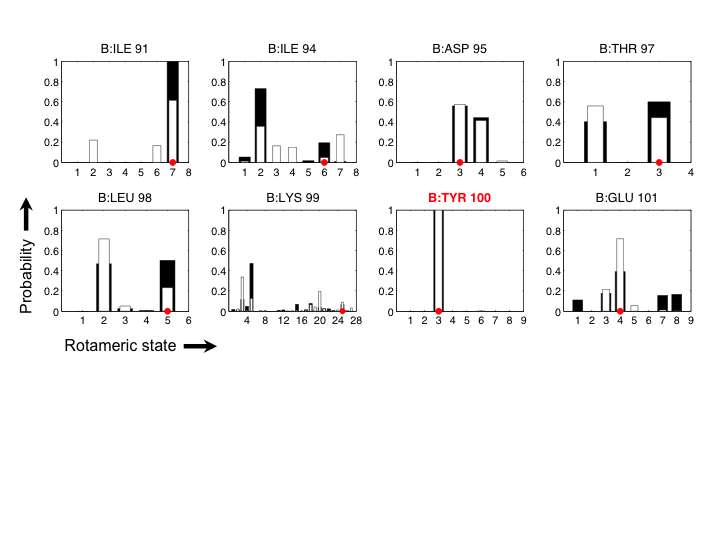

Supplement: S14 Fig — For details, see caption to S1 Fig. (TIFF) [file pone.0170337.s014.tiff]

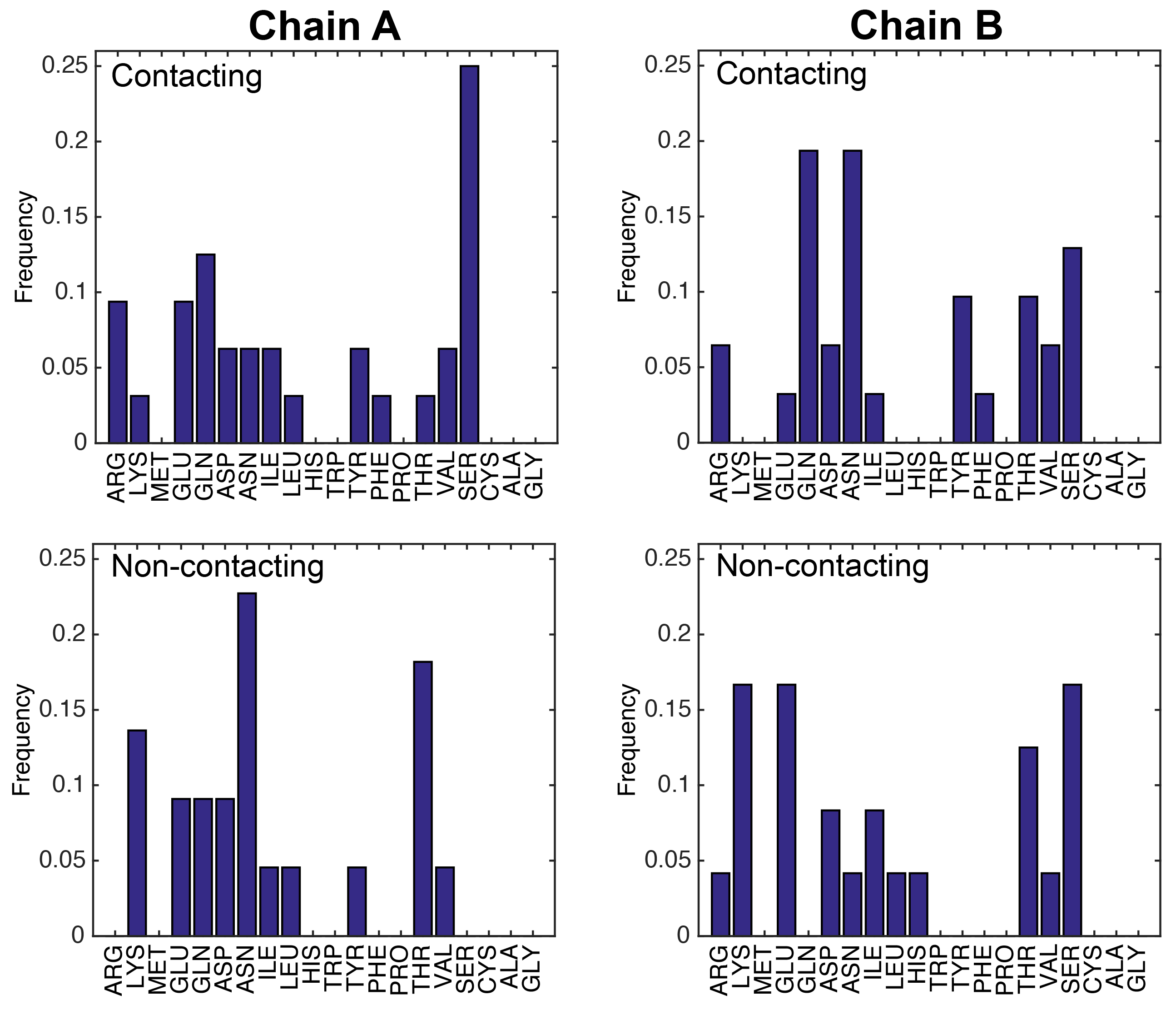

Supplement: S16 Fig — Frequency of residues in the contacting (top panels) and non-contacting (bottom panels) sets of residues in CVN chains A (at left) and B (at right). Residues on the horizontal axis are ordered based upon the number of rotameric states: left (more rotamers) to right (less rotamers) according to the Penultimate Rotamer Library [26]. Ala and Gly do not have any rotamers, and Cys is excluded from the analysis since the cysteins in CVN participate in disulfide bonds. (TIFF) [file pone.0170337.s016.tiff]

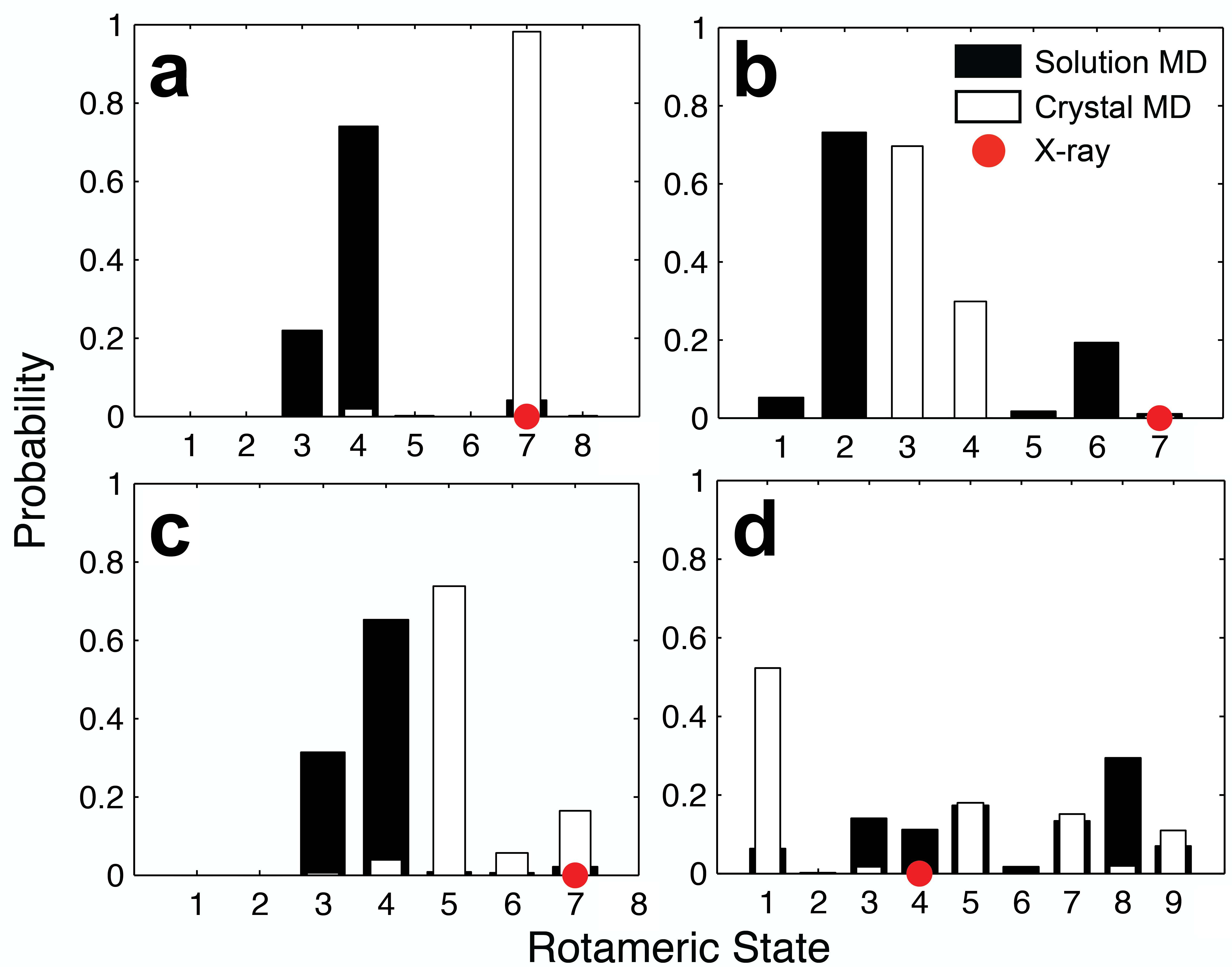

Supplement: S17 Fig — In the same manner as Fig 2 of the main text, rotameric states are denoted by numbers on the horizontal axis, and correspond to the order in which they appear in the Penultimate Rotamer Library [26] for each residue. Distributions obtained from solution and crystal simulation are shown with black and white bars, respectively, and the rotatmer observed in the X-ray structure is denoted by the red dot on the horizontal axis (legend in panel B). (a) Glu56 in chain A (A:Glu56, same as Fig 2C; OC = 0.06 and TΔSconf = –0.36 kcal/mol) is contacting, (b) A:Ile94 (OC = 0.01 and TΔSconf = –0.11 kcal/mol) is non-contacting, (c) B:Asn3 (OC = 0.08 and TΔSconf = –0.06 kcal/mol) is contacting, and (d) B:Gln14 (OC = 0.38 and TΔSconf = –0.44 kcal/mol) is contacting. (TIF) [file pone.0170337.s017.tif]
