# Supplementary material for: Effect of the Crystal Environment on Side-Chain Conformational Dynamics in Cyanovirin-N Investigated through Crystal and Solution Molecular Dynamics Simulations
Source: PLoS One. 2017 Jan 20;12(1):e0170337. doi: 10.1371/journal.pone.0170337 (PMC5249168; doi:10.1371/journal.pone.0170337)
Supplement: S1 Table — Side chains in simulation and experiment are determined to be in agreement if one of the two most dominant rotamers from simulation matches the X-ray conformation. Percent agreement is averaged over all solvent-exposed rotameric residues (“all”) and for the subsets of contacting (“cont”) and non-contacting (“non-cont”) solvent-exposed residues. For residues with alternate conformations in the crystal structure, conformation A was used for comparison. (DOCX) [file pone.0170337.s018.docx]

**Table S1: Agreement between dominant rotameric states from MD and X-ray for solvent-exposed residues in CVN**

|  | **Solution MD vs. X-ray** | | | **Crystal MD vs. X-ray** | | |
| --- | --- | --- | --- | --- | --- | --- |
| **chain^a^** | **all** | **cont** | **non-cont** | **all** | **cont** | **non-cont** |
| A | 0.59 | 0.72 | 0.41 | 0.72 | 0.84 | 0.54 |
| B | 0.64 | 0.58 | 0.71 | 0.78 | 0.84 | 0.71 |

^a^In chains A/B, at total of 54/55 residues are solvent-exposed, of which 32/31 are contacting and 22/24 are non-contacting.
